# Using an Instrumented Hammer to Predict the Rupture of Bone Samples Subject to an Osteotomy

**DOI:** 10.3390/s23042304

**Published:** 2023-02-18

**Authors:** Manon Bas dit Nugues, Giuseppe Rosi, Yoann Hériveaux, Guillaume Haïat

**Affiliations:** 1 Laboratoire Modelisation et Simulation Multi Echelle, Centre National de la Recherche Scientifique, MSME UMR 8208 CNRS, 61 Avenue du General de Gaulle, 94010 Creteil, France; 2 Laboratoire Modelisation et Simulation Multi Echelle, Universite Paris Est Creteil, MSME UMR 8208 CNRS, 61 Avenue du General de Gaulle, 94010 Creteil, France

**Keywords:** osteotomy, impact hammer, bone, biomechanics, fracture

## Abstract

Osteotomies are common procedures in maxillofacial and orthopedic surgery. The surgeons still rely on their proprioception to control the progression of the osteotome. Our group has developed an instrumented hammer that was shown to provide information on the biomechanical properties of the tissue located around the osteotome tip. The objective of this study is to determine if this approach may be used to predict the rupture of a bone sample thanks to an instrumented hammer equipped with a force sensor. For each impact, an indicator *τ* is extracted from the signal corresponding to the variation of the force as a function of time. A linear by part regression analysis is applied to the curve corresponding to the variation of *τ* as a function of the distance *d* between the tip of the osteotome and the end of the sample. The experiments were conducted with plywood and bovine trabecular bone samples. The results show that *τ* starts increasing when the value of *d* is lower than 2.6 mm on average, which therefore corresponds to a typical threshold detection distance between the osteotome tip and the sample end. These findings open new paths for the development of this instrumented surgical hammer.

## 1. Introduction

Osteotomies are surgeries consisting of cutting bone and cartilage tissues using a surgical instrument, named osteotome, which is impacted using a mallet. This type of intervention is performed in many surgical fields. In plastic and maxillofacial surgery, osteotomies are used for instance in rhinoplasties [1,2] as well as for instance in Summer’s osteotomies [3,4] which aim at improving bone density before dental implant surgery. Another common maxillo-facial surgery intervention where osteotomies are employed is the pterygomaxillary disjunction [5], which consists of cutting a bone plate (Lefort osteotomy) located under the jaw: the pterygo-palatine fossa. Osteotomies can also be used to remove bone grafts from the calvaria [6,7,8]. In orthopedic surgery, tibial valgus osteotomies procedures aim at restoring the lower limb alignment, in particular for patients with early knee osteoarthritis [9]. Eventually, vertebral body osteotomies are performed to treat spinal deformities such as scoliosis [10,11].

Despite their routine clinical use, osteotomies are often performed without any visual control and surgeons rely on empirical methods such as their proprioception to impact the osteotome and guide it in the desired path throughout bone tissue. In particular, a common feature of many osteotomies lies in the fact that the rupture of the bone sample should not be complete and should not lead to two separated bone samples. Instead, the crack tip should stop before the end of the sample to create a hinge that will be used to shape the sample as planned. Therefore, osteotomies are delicate surgeries with a long learning process, and failures may be associated with dramatic life-threating consequences. 

Different approaches have been developed to assist the surgeon during an osteotomy. Three-dimensional localization procedures coupled with X-ray-based imaging methods have been developed to track the position of the osteotome [12,13]. However, these tools are not used in clinical routines because of (i) increased operating time and cost and (ii) they are radiative for the patient. A piezoelectric cutting system named piezotome [14,15,16] has been developed and is used in some cases but remains difficult to use because it significantly modifies the surgical protocol.

An alternative method was developed by our group in order to provide feedback to the surgeon during an osteotomy. It consists of an instrumented hammer which had previously been developed to help the surgeon insert an acetabular cup [17,18,19,20,21] and femoral stem implants [22,23,24,25] into the host bone. The principle of the measurement is to record the signal corresponding to the time variation of the force applied between the hammer and the osteotome during an impact. An indicator is derived from each signal corresponding to each impact, which provides information to the surgeons on the mechanical properties of bone tissue located around the osteotome tip. The instrumented hammer is employed similarly as in the clinical practice so that this method does not modify the surgical protocol.

A feasibility study [26] has been performed using different materials including trabecular bone and plywood, showing that it is possible to retrieve information on the biomechanical properties of the material located around the osteotome tips. This study [26] was carried out by slightly impacting the osteotome without modifying its position throughout the impacts. It proved that using the instrumented hammer, (i) the material of a sample could be predicted with an estimated 89% prediction performance and (ii) its thickness could be estimated with an error lower than 10%. Later, it was also shown that the instrumented hammer could be used to detect a change in the tissue material properties during a rhinoplasty performed in rabbits [27] using a machine learning algorithm. The method was eventually applied to an anatomical subject showing the feasibility of this approach in a configuration close to that of the operating room [28,29].

Despite the possibility of assessing the biomechanical properties of bone tissue located around the osteotome tip, it remains impossible to anticipate nor to predict the rupture of a sample (experiments described in [26] were made with a constant osteotome position), which would be very useful in order to secure the aforementioned osteotomies. The aim of the present study is to determine whether an instrumented hammer can be employed in order to determine when a bone sample is fractured by the osteotome and possibly to anticipate this rupture. To do so, the same experimental approach was applied first to plywood plates and then to bovine trabecular bone samples. The osteotome was impacted using the instrumented hammer until rupture and each signal corresponding to each impact was recorded and analyzed.

## 2. Materials and Methods

### 2.1. Experimental Measurements

**Figure 1:**
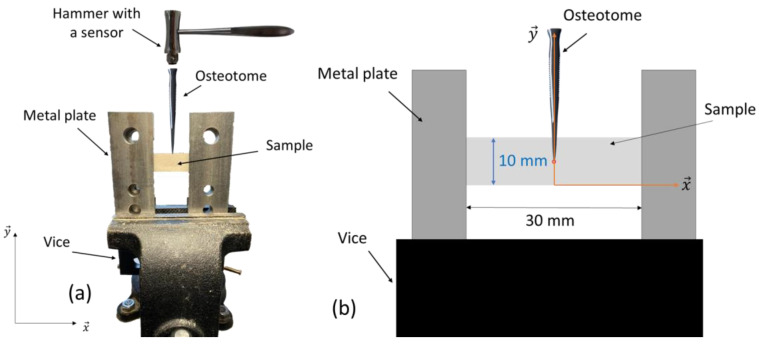
(**a**) Schematic representation of the experimental setup used for impaction of the osteotome on the plywood and trabecular bone samples. (**b**) Zoom showing the measurement of *d* on Tracker for a trabecular bone sample.

Two types of samples were considered. First, eight plates were cut from three-plies ordinary birch plywood. Second, twenty-two plates of trabecular bone were cut from four bovine femoral heads. Each plywood (respectively trabecular bone) plate had dimensions of around 70 mm × 15 mm × 3 mm (respectively 35 mm × 10 mm × 1.5 mm), which was measured with a digital caliper with an incertitude of around 0.03 mm. As shown in Figure 1, the samples were placed between four metallic plates, two at each end. The metallic plates were always placed perpendicular to the jaws of the vice. The sample was placed perpendicular to the metallic plates. The jaws were tightened in the same way each time. A compromise was found to determine the force applied to the metal plates so that (i) it is strong enough to avoid any movement during the experiment and (ii) it does not damage the sample. The torque applied to the vice handle to tighten the jaw was equal to around 117.75 ± 2.64 N/m. The plane of the sample *(x,y)* in Figure 1, matches the transverse anatomical plane of the bovine femoral bone samples. The osteotome therefore cuts the trabecular bone samples perpendicular to the loading axis of the bovine femur.

**Figure 2:**
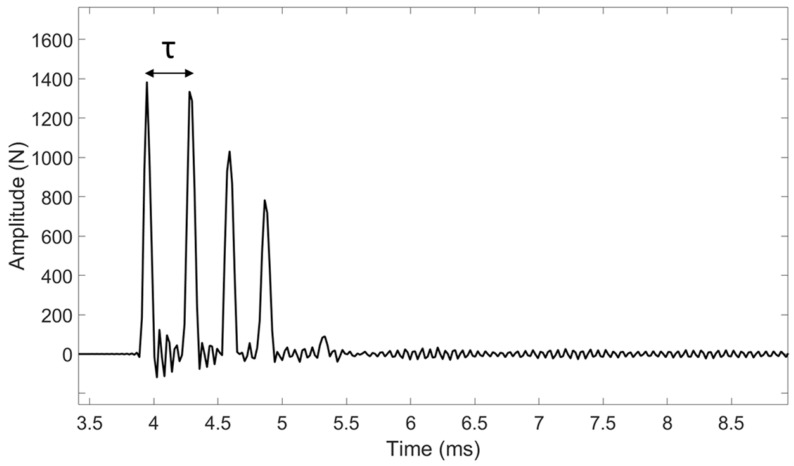
Example of a signal *s(t)* corresponding to the variation of the force as a function of time obtained for a given impact of the instrumented hammer on the osteotome with a plywood sample.

**Figure 3:**
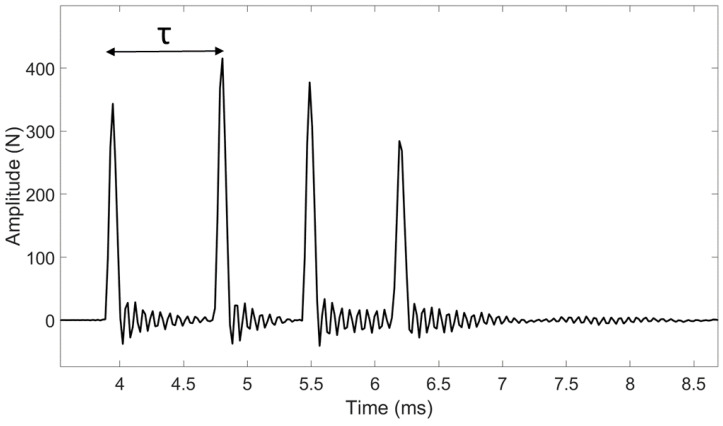
Example of a signal *s(t)* corresponding to the variation of the force as a function of time obtained for a given impact of the instrumented hammer on the osteotome with a trabecular bone sample.

The experimental measurements were performed similarly as in [26], except that the impacts were made with a higher energy in order to allow the progression of the osteotome in the sample. An osteotome used clinically (32-6002-10, Zepf, Tuttlingen, Germany) having a 10 mm-long cutting edge was used to cut each sample along its entire length. To do so, the osteotome was impacted with a 260 g surgical mallet (32-6906-26, Zepf, Tuttlingen, Germany) equipped with a dynamic piezo-electric force sensor (208C04, PCB Piezotronics, Depew, NY, USA). This sensor, which has a measurement range up to 4.45 kN in compression, was screwed in the center of the impacting face of the hammer. The time variation of the force was recorded by a data acquisition module (NI 9234, National Instruments, Austin, TX, USA) with a sampling frequency of 51.2 kHz and a resolution of 24 bits and transferred to a computer using a LabVIEW interface (National Instruments, Austin, TX, USA) for a duration of 2 ms. For each impact, the time dependence of the force applied to the osteotome was measured, leading to a radio frequency signal denoted *s(t)*. An example of typical signals obtained for each sample is shown in Figure 2 and Figure 3.

During all experiments, the position of the osteotome tip was assessed using a video camera (L-920M3, Spedal, Taiwan) positioned perpendicularly to the plane of the sample. This camera has a resolution of 1920 × 1080 pixels: the image has 1920 pixels in the x-direction and 1080 pixels in y-direction. The shape and size of the image sensor, named “image sensor format” is 1/2.7″, which corresponds to an area of the sensor of about 21.7 mm^2^. The framerate of the sensor is 30 frames per second (fps) and the ratio between the brightest and darkest parts of an image, named “imaging dynamic range” is 75 db. For each plate, the following steps were applied to extract d from the video. First, an image of the video was made after each impact of the hammer. Second, a tracker software (Tracker, version number 5.1, National Science Foundation, Alexandria, VA, USA) was used in order to estimate the value of *d* after each impact, with an inter-operator reproducibility estimated to around 0.13 mm. The origin of the *y*-axis corresponds to the bottom of the plate so that the last value of *d* was always negative when the recording was stopped since the osteotome crossed the plate edge, which corresponds to a complete rupture of the sample. No vertical guidance system was used for the osteotome since the surgeon was required to perform the osteotomy similarly as in the clinics. We performed random measurements of the angle between the axis of the osteotome and the *y*-axis to ensure that the osteotome progressed vertically and the maximum angle found was 6.7°. Third, the width of the plate in the y-direction was used as the image scale in order to convert pixel and measurements and to determine the values of *d*, which was carried out for each image taken between each impacts. For all samples, the last value of *d* was negative because the recording was stopped once the osteotome crossed the plate end, which corresponds to a complete rupture of the sample.

The osteotome was held manually similarly as what is performed in the clinic and it was impacted with a maximum force of 500 N and 1500 N for bone and plywood samples, respectively, which corresponds to a compromise between an impact energy (i) sufficiently low to avoid inducing a complete fracture of the sample and (ii) sufficiently high to induce a progression of the osteotome in the sample. 

### 2.2. Signal Processing

A dedicated signal processing technique illustrated in Figure 2 and Figure 3 was applied to each rf signal *s(t)* using information derived from the different peaks obtained in the signal, similarly as in previous studies from our group [26,27,28]. The time of the maximum of the first two peaks of *s(t)* was determined for each impact. The indicator *τ* was defined as the difference between the times of the second and first peaks of *s(t)*. 

### 2.3. Data Analysis

The variation of the indicator *τ* as a function of the distance *d* between the position of the osteotome tip and the end of the sample could be divided into three regimes. First, the value of *τ* decreases when *d* starts decreasing. Second, *τ* reaches an approximately constant value and third, *τ* increases when *d* reaches values close to 0. Here, we assumed a linear dependence of *τ* as a function of *d* for each of the three regimes, so that the relation between *τ* and *d* could be interpolated using Equation (1), where *d_i_* and *d_f_* are the values of the distance *d* delimiting the three different regimes, *k_i_* and *k_f_* are the slopes of the curve representing the variation of *τ* as a function of *d* during the initial and final regimes, respectively, and *τ_0_* is the value of *τ* at the plateau (second regime).

(1) $$\tilde{\tau }\left(d\right)=\{\begin{array}{c} \tau_{0}+k_{i}\times \left(d-d_{i}\right)\;if\;d\geq d_{i\;}\\ \;\tau_{0}\;if\;d_{f}\leq d\leq d_{i}\\ \;\tau_{0}+k_{f}\times \left(d-d_{f}\right)\;if\;d\leq d_{f}\; \end{array}$$

Based on the experimental results, a cost function *e_τ_ (d_i_, d_f_, k_i_, k_f_, τ_0_)* was defined for each plywood and bone sample in order to assess the difference between the experimental measurements and values given by Equation (1) following:

(2) $$e_{\tau }\;\left(d_{i},d_{f},k_{i},k_{f},\tau_{0}\right)=\overset{N}{\underset{j=1}{\sum }}\frac{\left|\tau \left(j\right)-\tilde{\tau }\left(d(j)\right)\right|}{N},\;$$

where *j* corresponds to the number of the impact, *N* to the total number of impacts for the sample considered, *τ(j)* corresponds to the value of *τ* for the impact *#j* and *d(j)* corresponds to the value of *d* for the impact *#j*. Note that N varies between 29 and 145 for the plywood samples and between 27 and 112 for the bone samples. An optimization procedure based on a conjugate gradient method was carried out in order to determine the optimal values of the parameters (*d_i_, d_f_, k_i_, k_f_, τ_0_*) minimizing the cost function *e_τ_* for each sample. The minimum value of *e_τ_* obtained by the optimization procedure is noted *E_τ_*.

## 3. Results

### Experimental Results

**Figure 4:**
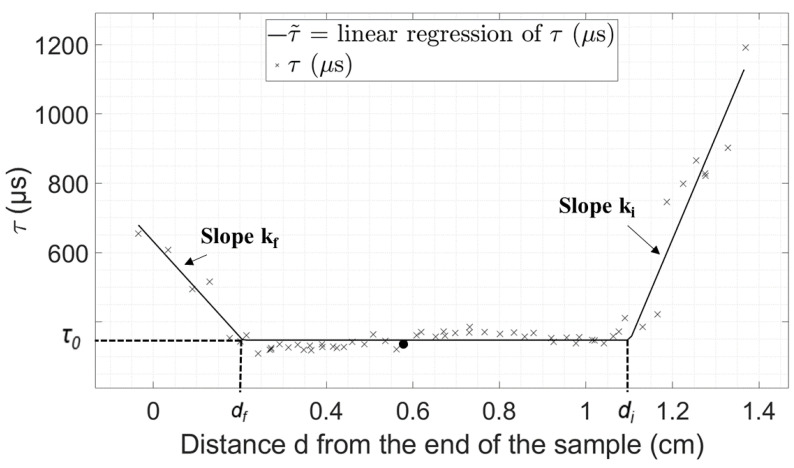
Variation of *τ* as a function of the distance from the end of the sample *(d)* obtained with a plywood sample. The linear by part interpolation $\tilde{\tau }$ obtained using the minimization procedure is shown by the solid line. The signal indicated by a circle is the signal shown in Figure 2.

**Figure 5:**
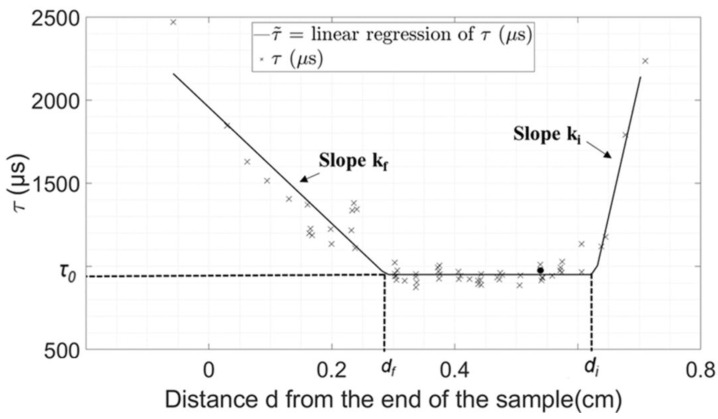
Variation of *τ* as a function of the distance from the end of the sample (*d*) obtained with a trabecular bone sample. The linear by part interpolation $\tilde{\tau }$ obtained using the minimization procedure is shown by the solid line. The signal indicated by a circle is the signal shown in Figure 3.

Figure 4 and Figure 5 show the evolution of *τ* as a function of *d* obtained for a given plywood and bone sample, respectively. The three different regimes described in Section 2.3 can be identified through the linear regression represented with solid lines, which corresponds to the results of the interpolation procedure described in Section 2.3. The results show a good agreement between the values of *τ* and its interpolation. Table 1 shows the values of all the parameters of interest (*τ_0_, d_i_, d_f_, k_i_, k_f_*) as well as the minimum value of *e_τ_* (noted *E_τ_*) corresponding to the results shown in Figure 4 and Figure 5 obtained using the minimization procedure described above.

The results obtained with the other plywood and bone samples were qualitatively similar to the ones shown in Figure 4 and Figure 5. Table 2 and Table 3 show the average, minimum, maximum values and standard variation of parameters *τ_0_*, *d_f_*, *k_f_*, *k_i_* and *E_τ_* describing the evolution of *τ* as a function of *d*. Table 2 and Table 3 show that the dispersion of the results is lower for the plywood samples than for trabecular bone, which may be explained by the dispersion of material properties obtained for the various bone samples, which is much lower for the plywood samples. Interestingly, the values of *k_f_* are always negative, which indicates a systematic increase of *τ* when the osteotome reaches the end of the plate.

## 4. Discussion

The use of an instrumented hammer in osteotomies had already been studied by our group in the context of rhinoplaties [27,28]. In these previous studies, the same hammer was used to detect crack propagation and the change of material properties at the tip of the osteotome. The main originality of the present study is to show that an instrumented hammer can be used to predict when the sample may be fractured by an osteotome. 

### 4.1. Repeatability of the Measurements

In order to assess the repeatability of the fastening method, a plywood sample was measured ten times during the stationary regime, then repositioned in the set-up and then measured again 10 times. All measurements were made with light impacts in order to avoid the osteotome progressing, which would modify the measurements. The average and standard deviation of the measurements obtained the first and second time were equal to 333.0 ± 13.6 µs and 323.4 ± 6.8 µs, respectively. An ANOVA analysis was performed (*p* = 0.012, F = 7.01), which indicates that repositioning the sample has an effect on the value of *τ*, even if this effect is moderate. However, we did not reposition the sample in the vice during the experimental protocol, so that the repeatability of the fastening method affects the reproducibility of the measurements between the samples, but not the conclusions obtained herein because we do not compare the results obtained with the different samples. Moreover, the difference induced by the fastening method is much lower than the standard deviation obtained for the values of *τ_0_* (see Table 2), which corresponds to an indicator of the intersample reproducibility.

The effect of the cables on the value of *τ* was investigated in a given configuration by comparing 10 values of *τ* obtained with four different cables with different properties. For each cable, the average and standard deviation of the values of *τ* is shown. We found that the cable significantly affects the results, which constitutes a limitation. However, the same wire was used for all measurements. The device described herein will have to be modified to be used in a clinical environment. In particular, it will need to be wireless, so that it does not affect the surgical protocol. However, the hammer used in this study is a prototype. Eventually, the information should be provided to the surgeons swiftly and in real time, which could be performed with the help of a visual and/or auditory indicator. For example, a light changing from green to red would indicate a rise in *τ* and would warn the surgeon that the osteotome is approaching the end of the sample.

### 4.2. Physical Interpretation of the Indicators

The behavior of the force as a function of time shown in Figure 2 and Figure 3 is in qualitative agreement with the results obtained in the context of rhinoplasty [27]. This behavior was explained by an analytical model [26] where the osteotome was modeled in 1D by a spring and the sample by a spring/dash model. The signals shown in Figure 2 and Figure 3 may be explained qualitatively by a rebound of the osteotome between the sample and the hammer during each impact. The analytical model developed in [26], as well as the experimental results, showed that *τ* is related to the rigidity of the sample located around the osteotome tip. An increase in the sample rigidity leads to an increase in the resonance frequency of the system, which leads in turns to a decrease in the value of *τ*. Such interpretation may help to understand the results obtained herein, as described below. 

Figure 4 and Figure 5 shows that three regimes may be distinguished to describe the behavior of *τ*. First, an initial decrease in *τ*, which is illustrated by the positive values of *k_i_*, is obtained for all samples. This decrease in the value of *τ* during the insertion of the osteotome may be explained by an increase in the amount of material in contact with the osteotome, which leads to an increase in the system rigidity. Second, a plateau is reached by the value *τ* (at a value *τ_0_* and between *d_i_* and *d_f_*), which may be explained by the fact that the contact conditions of the material around the osteotome become constant, while the osteotome is sufficiently far from the sample end to behave as a semi-infinite medium. Third, an increase in the value of *τ* is obtained when the osteotome approaches the end of the sample (for a distance lower than *d_f_* from the sample end). This increase in *τ* may be due to the loss of rigidity due to the decrease in the amount of material in front of the osteotome, leading to higher values of *τ*, as described in the analytical model [26]. 

### 4.3. Parameters Derived from Data Analysis

The first parameter of interest is the value of *τ_0_*, which corresponds to the constant phase of *τ.* The value of *τ_0_* depends on the mechanical properties of the material in which the osteotome tip is located [26]. Note that the mean value of *τ_0_* for the plywood samples in this study is comprised between 347.7 and 480 μs, while it was around 230 μs in our previous article [26]. This difference may be explained by the fact that the plates used herein are slightly longer (along *x*-axis) than the one used in [26]. Note that we checked that using plates with similar dimensions as the ones used in [26] led to similar results. 

Here, we found that the values of *τ_0_* obtained for the plywood samples are lower than for the trabecular bone samples. This result may be explained by the fact that the difference of Young’s modulus between the two materials. In order to quantify this difference, compression tests were performed with two bone samples coming from the femoral heads from which the plates were extracted. The Young’s modulus of bovine trabecular bone obtained using these tests are equal to 117 ± 9.42 MPa and 92.7 ± 1.47 MPa for the two samples considered. Please note that these values are of the same order of magnitude compared to those found in the literature for the femoral condyle (117.49 ± 61.53 MPa) [30] and for L3 vertebral body (173 ± 97 MPa) [31] extracted from calf. Even though the values extracted from the literature does not concern the same anatomical site, it confirms that calf trabecular bone young modulus is in the range of a hundred Mega Pascals. These values are considerably lower than the Young’s modulus of plywood (in the range of GPa according to [32]). Since *τ* is related to the mechanical properties of the material and in particular to the stiffness, the higher value of *τ_0_* obtained for trabecular bone compared to plywood is consistent with the difference of Young’s modulus. Another reason for this difference may also be related to the size of the samples which are not the same for bone and plywood samples.

The second parameter of interest is the value of *d_f_*, which corresponds to the distance between the end of the sample and the osteotome tip from which *τ* starts to increase. The parameter *d_f_* therefore corresponds to the typical distance between the osteotome tip and the end of the sample that can be detected by the method in order to warn the surgeon the sample may break. Interestingly, the average value of *d_f_* is similar for bone and plywood samples (~2.6 mm).

### 4.4. Limitations

This study has several limitations. First, the geometry of the samples is simplified compared to the various configurations of interest in the clinics. Future studies should be conducted in situations closer to the ones encountered in the clinics, which may be achieved using animal models and anatomical subjects [33]. However, it is a requirement for ethical issues to establish a proof of concept under controlled conditions before working with anatomical subjects. Another potential issue could be that the human samples may be composed of both trabecular and cortical bone and surrounded by soft tissues, which may also modify the results. However, it was shown in a previous study considering an instrumented hammer for the insertion of the acetabular cup implants that the presence of soft tissue did not modify the results [34]. 

Second, we did not consider crack initiation nor propagation in this simple situation because our aim was to determine whether it was possible to detect the end of the sample in a controlled situation. However, cracks may appear during rhinoplasty [35]. We have shown in previous studies corresponding to rhinoplasty [27,28,29] that this crack in front of the osteotome tip can be detected by a sudden decrease in the value of *τ* due to a decrease of the sample rigidity. Therefore, further work is needed to distinguish situations where a decrease in *τ* is due to (i) the osteotome reaching the end of the sample and (ii) the creation of a crack around the osteotome tip. 

Third, the results may depend on the geometry of the osteotome and in particular its length, material and width, which are parameters likely to affect its overall rigidity, which has been shown by the analytical model [26] to affect the response of the system. Future studies should take into account the osteotome type and assess the effect of modifying this parameter.

Fourth, the material of the sample as well as its size affect the results obtained herein. The effect of the material was investigated in [26] when the osteotome does not progress in the sample. Here, we have investigated bone and plywood. The change of material does not affect the general behavior obtained in Figure 4 and Figure 5. However, a change of material affects the values of *d_i_, d_f_, k_i_, k_f_, τ_0_* as well as their distribution. Inter -individual differences might also change the relation between *τ* and *d*. These differences, which are quantified in Table 2 (respectively 3) for the plywood (respectively bone) samples, may be explained by two reasons. First, they may come from the experimental conditions (impact force, sample positioning, angle between the impact and the osteotome axis), that are not exactly similar for all samples. Second, they may be explained by the difference in terms of mechanical properties of the sample. Although the first aspect is valid for all samples, the second one only applies to the bone samples, which is why the results are shown to be much more scattered for the bone samples than for plywood. The variation of bone material properties can be explained by the inter-individual and anatomic variation of the bone mechanical properties. Despite these differences, *k_f_* is always negative, so that there is always an increase in *τ* before the exit of the osteotome, regardless of the bone’s mechanical properties. When the plate is larger (along *y*-axis), the stationary regime (the plateau) becomes longer. Changing the length (along *x*-axis) of the plate does not affect the results if the distance between the two metal plates holding the sample remains the same, i.e., approximately 30 mm. If the distance between the two metal plates decreases, *τ* decreases as indicated in Section 4.3. Increasing the sample thickness leads to an increase in its rigidity and therefore leads to an increase in *τ_0_*. This parameter has already been studied in [26], where different thicknesses were considered for six different materials. The value of *τ* increased as a function of the sample thickness for all six materials, which is due to the increasing rigidity of the sample (see Figure 6 of [26]).

Fifth, the surgeons used the hammer in the same way as what they do in the clinics, impacting the osteotome perpendicularly to its axis. However, errors of around up to 10° are always possible and this error was not monitored precisely. In order to investigate the effect of this angle on the value of *τ*, we asked the surgeon to impact the osteotome 25 times (for a given configuration where the osteotome does not progress) similarly as in the experiments and then with an angle of around 10°. We then compared the averaged value and standard deviation of *τ* for these two configurations. The averaged value is similar for the two configurations, while the standard deviation obtained is slightly higher when an angle of 10° is considered.

## 5. Conclusions

This study is the first one to study the progression of an osteotome in a bone sample and to show that the instrumented hammer may detect when the osteotome approaches the end of the sample. The results show that our approach may be used to detect the sample end at an average distance of 2.6 mm. Future studies should be carried out to exploit this result in each configuration of interest in orthopedic and maxillofacial surgeries. This study may pave the way towards the development of a medical device consisting of a patient specific decision support system to alert the surgeon before the hinge collapses. 

## Figures and Tables

**Table 1:** Values of *τ_0_*, *d_i_*, *d_f_*, *k_i_*, *k_f_* and E*_τ_* (the minimum value of *e_τ_*) corresponding to the linear by part regression analysis $\tilde{\tau }$ (see Equation (2)) obtained with the minimization procedure for the samples shown in Figure 4 and Figure 5.

Parameter	Plywood Sample (Figure 4)	Bone Sample (Figure 5)
*τ_0_* (μs)	348	951
*d_i_* (cm)	1.10	0.63
*d_f_* (cm)	0.21	0.29
*k_i_* (μs/cm)	2956	16,220
*k_f_* (μs/cm)	−1375	−3495
*E_τ_*	5.04	10.9

**Table 2:** Mean, minimum, maximum values and standard variation of parameters *τ_0_*, *d_i_*, *d_f_*, *k_i_*, *k_f_* and E*_τ_* describing the evolution of *𝜏* as a function of *d* for plywood samples.

Parameter	Mean (±SD)	Min	Max
*τ_0_* (μs)	384.17 ± 44.07	347.7	480.1
*d_f_* (cm)	0.267 ± 0.104	0.19	0.5
*k_i_* (μs/cm)	3482 ± 1950	1169	6805
*k_f_* (μs/cm)	−1201.4 ± 795.7	−2977.6	−562.9
*E_τ_*	4.61 ± 3.27	1.25	12.08

**Table 3:** Mean, minimum, maximum values and standard variation of parameters *τ_0_*, *d_i_*, *d_f_*, *k_i_*, *k_f_* and E*_τ_* describing the evolution of *𝜏* as a function of *d* for bones samples.

Parameter	Mean (±SD)	Min	Max
*τ_0_* (μs)	1128.1 ± 359.5	560.9	1937.6
*d_f_* (cm)	0.262 ± 0.127	0.060	0.543
*k_i_* (μs/cm)	8751 ± 12,726	1281	58,071
*k_f_* (μs/cm)	−8268 ± 4794	−16,840	−1067
*E_τ_*	41.36 ± 36.05	8.29	140.31

## Data Availability

The data are stored in the MSME laboratory.
